# Long-term effects of COVID-19 pandemic on physical activity and eating behaviour of the Italian population: a longitudinal study

**DOI:** 10.1007/s12020-024-03950-w

**Published:** 2024-08-23

**Authors:** Giuseppe Bifolco, Ludovica Cardinali, Edoardo Mocini, Mirko Duradoni, Carlo Baldari, Marina Ciampi, Silvia Migliaccio, Luisella Cianferotti

**Affiliations:** 1 Bone Metabolic Diseases Unit, Department of Experimental & Clinical Biomedical Sciences, University of Florence, Florence, Italy; 2 Department of Life Science, Health, and Health Professions, Link Campus University, Rome, Italy; 3 Department of Experimental Medicine, University Sapienza of Rome, Rome, Italy; 4 Department of Education, Languages, Interculture, Literatures and Psychology, University of Florence, Florence, Italy; 5 Department of Theoretical & Applied Sciences, eCampus University, Rome, Italy; 6 Department of Economic and Social Sciences, University Sapienza of Rome, Rome, Italy

**Keywords:** Covid-19, Online survey, Exercise, Sedentary behaviour, Eating habits, Mediterranean Diet

## Abstract

**Background:**

Restrictive measures due to the Covid-19 pandemic strongly impacted lifestyle and daily behaviour. The purpose of this longitudinal retrospective study was to investigate short-term and long-term effects of Covid-19 pandemic on physical activity and eating habits of the Italian population investigating three time periods: pre-, during- and post-lockdown.

**Methods:**

A sample of 2773 adults recruited through social media provided data by an online survey administered from July to October 2023. Participants completed the International Physical Activity Questionnaire–Short Form (IPAQ-SF), the Mediterranean Diet Adherence Screener (MEDAS) and provided information about eating habits, socio-demographic and anthropometric characteristics.

**Results:**

There was a significant increase (p < 0.001) in mean BMI from pre-pandemic period (24.53 ± 5.34 Kg/m^2^) to post-pandemic period (25.22 ± 6.0 Kg/m^2^). Physical Activity significantly decreased during the pandemic period compared to the pre-pandemic period (χ² = 271.97; p < 0.001; φ = 0.31) with an increase in inactive subjects from 25.7% to 52.8%. In the post pandemic period, there was an increase in the level of Physical Activity compared to the pandemic period (χ² = 413.61; p < 0.001; φ = 0.39) with a reduction of inactive subjects from 52.8% to 25.6%. Adherence to Mediterranean Diet score significantly (p < 0.001) increase from pre-pandemic (7.18 ± 1.58) to during-pandemic (7.29 ± 1.69) and post-pandemic (7.75 ± 1.63) periods with significant differences emerged in the consumption of single MEDAS items during the pandemic period by different BMI classes. Consumption of seasonal fruit and vegetables, water intake, the preparation/consumption of traditional or local dishes and the time dedicated for dinner and lunch significant increase (p < 0.001) during pandemic.

**Conclusions:**

The Covid-19 pandemic changed people’s lifestyles, but in different ways for Physical Activity and diet. During the pandemic there was a negative effect for PA that decreased while the time spent sitting increased. This seems to be a temporary effect as, after the end of the phase of mandatory restrictions, it returns to the original level. The lockdown period improved the quality of the Italian population’s eating habits, with an increase in adherence to the Mediterranean diet even after the end of the pandemic showing a rediscovery of traditional dishes, increase in consumption of seasonal products, greater preference for local products and more time spent preparing meals.

## Background

On March 11, 2020, the World Health Organization [WHO] declared the SARS-CoV-2 outbreak a global pandemic [1]. In response, countries worldwide implemented public health measures to prevent mass contagion [2]. These measures also referred to as non-pharmacological measures (NPMs), including personal protection (e.g., masks and hand hygiene), environmental (e.g., disinfection and ventilation), social distancing (e.g., school and workplace closures, banning large gatherings) and travel-related (e.g., travel restrictions) dramatically impacted daily behaviours [1–5]. Italy was the first European country to be severely hit by the Covid-19 epidemic [5]. In February 2020, local SARS-CoV-2 transmission clusters were identified in Northern Italy and progressively spread to central and southern regions of Italy as well [5]. On March 9th 2020 a national lockdown was ordered, involving the closure of all public places and a ban on gatherings with requirements for residents to stay within their home, suspension of common commercial activities, religious celebrations, and travel restrictions until the 4th of May 2020 [6]. Regular office work was suddenly discontinued for many workers and employers and it was replaced by working from home [6, 7]. Simultaneously, in-person teaching for students and exercise in gyms were suspended and replaced with remote digital technology [6, 8]. Despite the obvious benefits of social distancing measures in reducing the spread of SARS-CoV-2, they profoundly impacted citizens’ lives, notably altering eating habits and physical activity (PA) behaviour [9]. For some individuals, the increased time spent at home during COVID-19 isolation provided an opportunity to spend more time on self-care and improving their health, while for others it was a source of stress that caused deterioration in both physical and mental health [10]. Some studies have shown increased adherence to the Mediterranean Diet (MD) [11, 12], which is recommended as one of the best nutritional pattern for health benefits [13], due to the increased time available for healthy cooking, and an increase in PA due to reduced commuting time, especially in the morning hours [6, 12, 14]. In contrast to these findings, some studies have shown an increase in boredom, stress and other types of negative emotions that have led to an increased and consumption of unhealthy and stress-reducing “comfort foods”, ultra-processed foods especially sweets, snacks, and baked goods, snacking between meals, more main meals overall, and even binge eating disorders [4, 15–18]. In addition to the change in eating habits, there was a reduction in PA and an increase in sedentary behaviour related to the closure of gyms and public parks. This shift encouraged excessive time spent at home in stationary positions, watching television or using mobile devices [4, 15]. Moreover, the increase in remote work and study from home has led to a reduction in daily energy expenditure and an increase in time spent sitting [19]. From May 4, 2020 until August 6, 2021, a series of containment measures followed, alternating with other lockdown periods until March 31, 2022 when the end of the covid-19 state of emergency in Italy was declared. The gradual reactivation of normal activities revealed the need to cope with new routines and behaviours that in some cases resulted in positive changes to healthy behaviours while in others resulted in negative changes [6, 20]. Most of the studies have evaluated the short-term effects of lockdown and restrictive measures on PA and eating habits while few studies [21] have examined the long-term impact of restrictive measures that could compromise health. Consequently, it is not entirely clear whether the changes in diet and PA behaviours due to the restrictions were only short-term or whether people permanently changed their behaviour positively or negatively.

Therefore, the aim of this study was to evaluate the impact of lockdown periods and long-term effects of restrictive measures on eating habits, adherence to MD and PA in the Italian population through the use of a questionnaire administered after the lockdown periods and which investigated retrospectively three periods: the pre-pandemic period (before January 2020), the national stay-at home lockdown period (March 9th - May 3th 2020) and post pandemic period (after April 2022, end of covid-19 state of emergency in Italy).

## Methods

### Population and study design

The study protocol involved the assessment of eating habits, adherence to MD and PA investigated retrospectively at three-time points: (i) before the pandemic lockdown, (ii) during the first pandemic lockdown (specifically during the period of the first lockdown confinement, March 2020-May 2020), (iii) after the pandemic lockdown period (after April 2021, i.e., after the end of the 3 subsequent lockdowns). The target population consisted of adults (> 18 years or older) with or without comorbidities. The self-administered questionnaire was designed by a multidisciplinary group that included public health specialists and was uploaded and shared on the Google Forms platform. The same questionnaire could only be completed once. Duplicate data were excluded through an e-mail detection (but not registration) system. The questionnaire was developed by the primary investigators following a review of related literature. It was evaluated and assessed by several experts, and specific modifications were made where needed. In addition, the questionnaire was shared as a pilot-test version among (n = 7) participants to check clarity and the suitability of wording, as well as the average time needed for its completion. The link to the electronic questionnaire was distributed through various social media: Instagram, Facebook, WhatsApp, LinkedIn and personal contacts among the members of the research group. The survey was active from July 18th, 2023 to October 6th, 2023. To limit dropouts, the questionnaire had a maximum duration of 10–15 min, allowing a better adherence to the study. Before starting the questionnaire, the participants received a brief description of the survey and its intent, the study protocol and the declaration of anonymity and privacy. During the survey, participants were able to stop study participation and leave the questionnaire at any section before the submission process; in doing so, their responses would not be saved. Responses were saved only by clicking on the “send” button provided at the end of the questionnaire. The participants were not compensated in any way for taking part in the study. The present study complied with the principles of the Helsinki Declaration, and all participants provided written informed consent to participate in the study and publication of the data. The study was approved by the Research Ethics Committee at e-Campus University (approval number: 07/2023).

### Survey questionnaire

The questionnaire included 46 items and it was divided into three sections:

socio-demographic and anthropometric information: age, gender, marital status, children, education level, region of residence. Self-reported anthropometric measurements include weight and height.
(2) International Physical Activity Questionnaire short form (IPAQ-SF).
  PA was assessed with the validated IPAQ-SF [22] that measures the type and amount of PA. The questionnaire consists of seven items investigating the amount (days per week and minutes per day) of vigorous activities (e.g., heavy lifting, heavy work in the garden, aerobic activities such as running or bicycling at high speed), moderate activities (e.g., carrying light weights, bicycling at regular speed, gym activities, garden work, prolonged physical work at home), walking activities, and time (minutes per day) spent in a sedentary position. To calculate the score, the Metabolic Equivalent of Task (METs) were calculated as: (i) number of days in which vigorous PA took place x minutes spent on vigorous activity x 8; (ii) number of days during which moderate PA took place x minutes spent on moderate activity x 4; (iii) number of days walked x minutes spent walking x 3.3. Total METs were calculated as the sum of Walking, Moderate, and Vigorous MET scores. If total METs were ≤700, the subject was classified as inactive, for scores between 700 and 2519 the subject was classified as moderately active, and for score ≥ 2520 the subject was classified as active or very active. The sitting question was developed as a separate indicator and not as part of the summed PA score.
Mediterranean Diet Adherence Screener (MEDAS)

Adherence to a MD was assessed by the MEDAS questionnaire from the PREvención con DIeta MEDiterránea (PREDIMED) study, a primary prevention nutritional intervention trial [23]. MEDAS is a 14-point questionnaire, which includes 12 questions on food consumption frequency and 2 questions on food intake habits related to the MD. A value of 0 or 1 was assigned to each question; the value 1 was applied when the adoption of the MD was met, while the value 0 was assigned when the condition was not met. Briefly, one point was scored when participants selected olive oil for cooking, daily consumption of four or more tablespoons of olive oil, white meat vs. red meat, two or more servings of vegetables, three or more pieces of fruit, less than one serving of red meat, hamburgers, sausages or deli meats, less than one serving of carbonated or sugary drinks, weekly intake of seven or more glasses of wine, three or more servings of legumes, three or more servings of fish/seafood, three or more servings of nuts, less than two servings of non-homemade pastries, white meat such as turkey or chicken preference instead red meat and two or more dishes seasoned with tomato, garlic, onion or leeks and sautéed with olive oil (“soffritto”). The MEDAS score (sum of the above items) ranged between 0 and 14. Based on the MEDAS score, participants were divided into three classes: low (score ≤ 5), moderate (score between 6 and 9), and high (score ≥ 10) adherence to the MD. This questionnaire was integrated with a specific question on water consumption. Participants were also asked to answer six items aimed at investigating changes in their general dietary habits during the lockdown, including time of cooking lunch and dinner, meal consumption in company, consumption of 0-kilometer food products, preparation of traditional or local recipes and choice of seasonal products.

All questions were designed to assess whether participants increased, decreased or maintained their habits across the lockdown period.

### Data analysis

For statistical analysis, the statistical package for social sciences (SPSS Inc., Chicago, IL, U.S.A.) was used (Statistical Package for Social Sciences software, version 19). Categorical variables were presented using frequencies and percentages. Continuous variables with an approximate normal distribution are represented by means and standard deviations. The chi-square test for categorical variables and one-way analysis of variance for repeated measures (ANOVA) for continuous variables were used to examine group differences in the three pandemic-periods. In the case of non-normal distributed variables, we relied on Friedman’s ANOVA.

## Results

### Sample description

A total of 2773 adults from all Italian regions completed the survey. The mean age was 35.6 ± 9.85 years, with 4.8% male, 94.8% female and 0.4% others. Demographics of the participants are shown in Table 1.

**Table 1:** Demographics of study participants

Demographics	All participants (*n* = 2773)
Age (years)	35.6 ± 9.85
Gender	
Female	94.8%
Male	4.8%
Others	0.4%
Region	
Northern Italy	54.8%
Central Italy	26.1%
Southern Italy	19.1%
Education level	
Bachelor, PhD	71.6%
High School	27.3%
Primary education	1.0%
Marital Status	
Single	45.2%
Married	51.1%
Divorced	3.1%
Widowed	0.6%

Data are reported as mean (SD) or percentage %

### Social media platforms recruitment

Instagram was the most popular social media platform used for recruitment (98.3%), followed by Whatsapp (0.8%), Facebook (0.2%), and Linkedin (0.1%). While 0.6% of participants were recruited by oral communication.

### Body Mass Index (BMI)

**Fig. 1:**
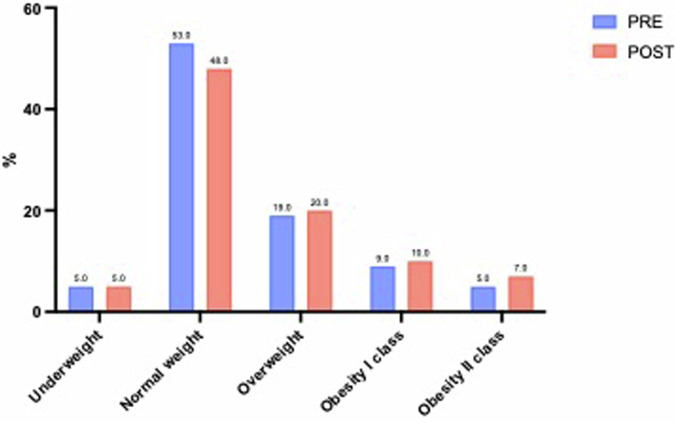
BMI categories distribution (in %) in pre and post pandemic periods

Self-reported data on height and body mass weight (before and post pandemic) were used to calculate BMI by using the Quetelet equation (body mass (kg)/height (m^2^)) and interpreted according to the criteria of the World Health Organization [24] resulting in five categories: underweight (BMI < 18.5 Kg/m^2^), normal weight (18.5 Kg/m^2^ ≤ BMI < 25.0 Kg/m^2^), overweight (25.0 Kg/m^2^ ≤ BMI < 30.0 Kg/m^2^), obesity category I (30.0 Kg/m^2^ ≤ BMI < 34.9 Kg/m^2^) obesity category II (35.0 Kg/m^2^ ≤ BMI < 39.9 Kg/m^2^). There was a significant increase (p < 0.001) in mean BMI from pre-pandemic period (24.53 ± 5.34 Kg/m^2^) to post-pandemic period (25.22 ± 6.0 Kg/m^2^) with 19% of participants classified as overweight in pre-pandemic compared to 20% in post-pandemic, 9% classified as obesity category I in pre-pandemic compared to 10% in post-pandemic and 5% classified as obesity category II in pre-pandemic compared to 7% in post-pandemic. The percentages of BMI categories are reported in Fig. 1. BMI showed a significant positive correlation with age (p < 0.001, r = 0.238) both in pre-pandemic and post pandemic periods with higher value in older than in younger participants, while gender was not significantly associated with BMI in either the pre-pandemic and post-pandemic periods.

In the pre-pandemic period, the normal weight category showed significantly higher levels (p < 0.001) in both MEDAS score [7.28] and PA levels (33.9% active/very active), while the obesity II category showed significantly lower levels in both MEDAS score [6.48] and PA levels (14.7% active/very active) than the other categories. This condition was maintained in the post-pandemic period for PA, with higher levels in the normal-weight category (33.0% active/very active) and lower levels in the obesity II category (23% active/very active), while the MEDAS score was significantly (p < 0.05) higher in the normal-weight category [7.84] and significantly lower in the obesity I category [7.56].

### Physical activity level

Statistical analysis reported a significant difference (p < 0.001) in PA level during the pandemic compared with the pre-pandemic and post-pandemic periods. No significant differences were found in PA level between pre and post pandemic. The participants’ level of PA was significantly reduced during the pandemic period compared to the pre-pandemic period (χ² = 271.97; p < 0.001; φ = 0.31). More specifically, there was a significant reduction in the number of active/very active subjects (during-pandemic: 20.9% vs pre-pandemic: 30.0%) and sufficiently active subjects (during-pandemic: 26.3% vs pre-pandemic: 44.4%), while the number of inactive subjects increased (during-pandemic: 52.8% vs pre-pandemic: 25.7%). In the post pandemic period, there was an increase in the level of PA compared to the pandemic period (χ² = 413.61; p < 0.001; φ = 0.39) in particular an increase in active/very active subjects (during-pandemic: 20.9% vs post-pandemic: 29.1%) and sufficiently active subjects (during-pandemic: 26.3% vs post-pandemic: 45.3%) while inactive subjects decrease significantly (during-pandemic: 52.8% vs post-pandemic: 25.6%). PA level variation over the study periods is reported in Table 2.

**Table 2:** Physical activity level before, during and post pandemic

	Pre-pandemic			During-pandemic			Post-pandemic		
**Inactive**	25.7%			52.8%			25.6%		
	M		F	M		F	M		F
	17.4%		26.1%	49.2%		52.9%	22.7%		25.7%
**Sufficiently active**	44.4%			26.3%			45.3%		
	M		F	M		F	M		F
	39.4%		44.6%	25.0%		26.4%	35.6%		45.9%
**Active/Very active**	30.0%			20.9%			29.1%		
	M		F	M		F	M		F
	43.2%		29.3%	25.8%		20.7%	41.7%		28.3%

*M* Male, *F* Female

The analysis showed a different level of PA between genders in pre-pandemic period (p < 0.001) and post-pandemic period (p < 0.05) while no differences were found during-pandemic period between genders. Age was negatively correlated with PA level before the pandemic (p < 0.001, r = −0.70), during the pandemic (p < 0.001, r = −0.100) and after the pandemic (p < 0.001, r = −0.110) showing higher levels in younger than in older participants. The questionnaire included a question about the time, in minutes, spent sitting during a day. Statistical analysis reported a significant increase (p < 0.001) in the mean number of minutes spent sitting per day during the pandemic (398.50 ± 191.07 min) compared with the pre-pandemic (309.60 ± 170.71 min) and post-pandemic (314.30 ± 175.29 min) periods, showing that home confinement caused an increase in time spent sitting, compared with the pre-pandemic and post-pandemic periods. However, a higher level of PA was significantly associated with a lower level of sedentariness in both pre-pandemic (p < 0.001, r = −0.092), pandemic (p < 0.001, r = −0.184) and post pandemic (p < 0.001, r = −0.122) periods.

### Adherence to mediterranean diet

Statistical analysis reported a significant (p < 0.001) increase in MEDAS mean score from pre-pandemic (7.18 ± 1.58) to during-pandemic (7.29 ± 1.69) and post-pandemic (7.75 ± 1.63) periods. The different levels of adherence to the MD during the three periods are shown in Table 3. MEDAS score showed a significant positive correlation with age both in pre-pandemic (p < 0.001, r = 0.082), during pandemic (p < 0.001, r = 0.065) and post pandemic (p < 0.05, r = 0.037). No differences were found in MEDAS mean score between genders in all the three pandemic periods.

**Table 3:** Adherence to the Mediterranean diet during the three pandemic periods

Adherence to the MD	Pre pandemic n(%)	During pandemic *n*(%)	Post pandemic *n*(%)
Low	381 (13.7)	400 (14.4)	273 (8.5)
Moderate	2176 (78.5)	2097 (75.6)	2139 (77.1)
High	216 (7.8)	276 (10.0)	397 (14.3)

The positive criteria to the MEDAS questionnaire are reported in Table 4. Olive oil is preferred by the majority of the sample and its consumption increased significantly during the pandemic (p < 0.001), while there was a significant reduction (p < 0.001) in the post-pandemic period. The consumption of vegetables, fruit, legumes and nuts increases significantly from the pre-pandemic to the post-pandemic period (p < 0.001). The consumption of butter margarine or cream, sweet/carbonated beverages and sweets increased significantly during the pandemic period, while it decreased in the post-pandemic period (p < 0.001). As regards meat, consumption of red meat less than one serving a day decreased significantly from the pre-pandemic to the post-pandemic period (p < 0.001), however the number of people eating no meat increased considerably (F = 16.43; p < 0.001), while the preference for white meat over red meat increased significantly (p < 0.001) from pre-pandemic to post-pandemic period. The consumption of fish and shellfish significantly (p < 0.001) decreased during the pandemic period compared to pre and post pandemic. Wine consumption decreased significantly (p < 0.001) in post-pandemic compared to pre and during pandemic. Dishes seasoned with “soffritto” significantly increased during-pandemic (p < 0.001) while it decreases in the post-pandemic period (p < 0.001).

**Table 4:** Positive answer to MEDAS questionnaire

MEDAS item	pre-pandemic	during-pandemic	post-pandemic	*p*
	*n* (%)			
Olive oil, yes	69.3%	68.8%	72.5%	0.09
Olive oil, ≥ 4 ts/day	550 (19.8)	723 (26.1)	452 (16.3)	<0.001
Vegetables, ≥ 1 s/day	1720 (62.0)	1792 (64.6)	2070 (74.6)	<0.001
Fruits, ≥ 3 s/day	528 (19.0)	602 (21.7)	721 (26.0)	<0.001
Red meat, < 1 s/day	1671 (60.3)	1610 (58.1)	1596 (57.6)	<0.001
Butter, <1 s/day	2624 (94.6)	2434 (87.8)	2688 (96.9)	<0.001
Sweet beverage, < 1 s/day	2540 (91.6)	2381 (85.9)	2560 (92.3)	<0.001
Wine, 7 s/week	125 (4.5)	338 (12.2)	140 (5.0)	<0.001
Legumes, ≥ 3 s/week	570 (20.6)	770 (27.8)	1229 (44.3)	<0.001
Fish and seafood, ≥ 3 s/week	323 (11.6)	322 (11.6)	442 (15.9)	0.09
Sweets, < 3 s/week	1492 (53.8)	1163 (41.9)	1529 (55.1)	<0.001
Nuts, ≥ 3 s/week	573 (20.7)	683 (24.6)	842 (30.4)	<0.001
White meat over red	1922 (69.3)	1909 (68.8)	2010 (72.5%)	<0.001
“Soffritto”^a^ ≥ 2 s/week	1074 (38.7)	1262 (45.5)	916 (33.0)	<0.001

*ts* tablespoons, *s* serving. Data are expressed as number and percentage in parenthesis (*n* (%))

^a^traditional Italian mix of sauteed aromatic vegetables

Significant differences emerged in the consumption of single MEDAS items during the pandemic period by different BMI classes. Underweight class consumed mainly fruit (p = < 0.05) and nuts (p = < 0.05). Normal weight class consumed mainly vegetables (p = 0.05), wine (p < 0.001) and legumes (p < 0.001). Overweight class consumed mainly wine (p < 0.001). Obesity I class consumed mainly red meat (p < 0.001), olive oil (p < 0.001), butter, margarin or cream (p < 0.001) and sweet products (p = 0.05). Obesity II class consumed mainly olive oil, butter, margarin or cream (p < 0.001), sweet/carbonated beverages (p < 0.001).

### Eating habits

The Mediterranean model is also characterised by elements that go beyond food choice. Conviviality, consumption of seasonal foods and local products make the Mediterranean model unique and contribute to its value. The results show that from the pre-pandemic to the post-pandemic period, the percentage of people who always eat meals in company has decreased. During the pandemic there was a significant increase in consumption and choice of seasonal fruit and vegetables and a significant increase in water intake (p < 0.001); these behaviours that have improved further in the post-pandemic period (p < 0.001). Water intake was positively correlated with adherence to MD in pre-pandemic (p < 0.001, r = 0.198) during the pandemic (p < 0.001, r = 0.182) post-pandemic (p < 0.001, r = 0.160) and positively correlated with PA in pre-pandemic (p < 0.001, r = 0.134), during the pandemic (p < 0.001, r = 0.167), and post-pandemic (p < 0.001, r = 0.124). The preference of “zero-km-products” increased mainly in the post pandemic (p < 0.001). Moreover, during the pandemic period there was a significant increase in the preparation/consumption of traditional or local dishes (p < 0.001) and the time dedicated for dinner and lunch has increased significantly (p < 0.001), in the latter case, this habit remained even in the post pandemic.

### Correlation between physical activity and mediterranean diet

High adherence to the MD diet was positively correlated with a high level of PA both in pre-pandemic (p < 0.001, r = 0.176) during pandemic (p < 0.001, r = 0.182) and post pandemic (p < 0.001, r = 0.154) periods.

## Discussion

The present retrospective and longitudinal study was designed to examine the long-term effects of COVID-19 pandemic on the lifestyle of the Italian population. The main aim was to identify changes in PA participation, eating habits and adherence to MD in the Italian population at the beginning of the pandemic and to verify whether these returned to pre-pandemic levels with the end of restrictions. The results of this study showed that the COVID-19 pandemic and home confinement had a significant impact on the lifestyle habits of the Italian population, with different trends for PA compared with adherence to the MD and eating habits.

PA plays a crucial role in maintaining overall health and well-being and is associated with reduced risk of cardiovascular disease and metabolic disorders and lower overall mortality rates [25]. The first part of the questionnaire on PA levels revealed a decline in PA at the beginning of the pandemic with a significant reduction in both the number of active/very active from 30.0% before the pandemic to 20.9% during the pandemic, and the number of sufficiently active subjects from 44.4% before pandemic to 26.3% during the pandemic, while the number of inactive subjects increased from 25.7% before the pandemic to 52.8% during the pandemic. Several factors could explain these observations. First, the closure of gyms and swimming pools where people used to exercise [26] in addition to the prohibition of jogging, walking and cycling in parks and open place, prevented people from continuing their exercise. Moreover, despite the increase of online training which became the number one trend during the COVID-19 pandemic [27] and home-based exercise classes administered in both live streaming and pre-recorded modality through social media or smartphone apps [8], the lack of adequate equipment and space for PA could be another possible explanation for individuals’ inability to adequately maintain their normal PA at home [15, 19]. In addition, psychological distress resulting from the situation may have affected participation in PA. These results are consistent with previous literature studies [15, 19, 26] which reported a decrease in overall PA when comparing self-reported activity levels during and before home confinement. However, these changes were not persistent in the post-pandemic period. As expected, the results of our study showed an increase in PA level in the post-pandemic period compared to the lockdown period, with a significant increment in both the number of active/very active from 20.9% during the pandemic to 29.1% after the pandemic, and sufficiently active from 26.3% during the pandemic to 45.3% after the pandemic, while a decrease in inactive from 52.8% during the pandemic to 25.6% after the pandemic. This difference can be explained by an improvement in the situation due to the gradual relaxation of restrictive measures and a progressive return to normal activities. Another important finding of this study is the significant increase in time spent sitting per day during the pandemic (398.50 ± 191.07 min) compared with the pre-pandemic period (309.60 ± 170.71 min), demonstrating that home confinement caused an increase in sedentary lifestyle which could be due to the increase of sedentary jobs from home and consequently spending more hours in front of the computer [16]. Differently, after the pandemic the time spent sitting per day decreased significantly (314.30 ± 175.29 min) compared with the pandemic period (398.50 ± 191.07 min) without, however, returning to pre-pandemic level. This could be attributed to several factors, such as the change of working patterns toward a “working from home” modality as a result of COVID-19 pandemic. Indeed, previous studies [14, 28, 29] demonstrated that work from home is associated with high total sedentary behaviour. The results of the study showed a significant difference in PA level between genders both in the pre-pandemic and post-pandemic periods, with females being more inactive than males. However, during the pandemic period these differences did not emerge. This outcome is contrary to prior evidence which found that women were less active during lockdown [30] but in agreement with those obtained by Bu et al. [31] who found no gender differences between inactive, moderate active, and highly active classes. Moreover, people who were younger were more likely to be active than older participants in all study periods. In contrast, Qin et al. [30] showed that young adults aged 20–34 years had the higher prevalence of insufficient PA during COVID-19 home confinement.

Despite the phenomenon of globalisation and in recent years, a trend increasingly closer to the Western Diet, in Italy during the Covid-19 pandemic there was an improvement in eating habits with a consequent increase in adherence to the MD, defined as “moderate” by the MEDAS score. Result in agreement with the study by Di Renzo et al. and Izzo et al. [9, 32]. Several factors may have led to this effect, probably the reduction of frenetic rhythms, more time spent at home for focusing on meal planner and the various advertisements and campaigns on social media platform, such as the #eatwellcovid-19 [33] promoted by healthcare professionals incentivizing well-being and healthy habits. The international scientific community considers the MD to be a healthy and balanced eating pattern that can reduce the incidence of non-communicable diseases such as type 2 diabetes, cancer and cardiovascular diseases, and in improving the immune system [34]. Effectively, the anti-inflammatory properties of the MD and its nutritional profile rich in vitamins, monounsaturated fatty acids, and flavonoids can improve the immune system response and reduce the severity of COVID-19 [35]. For this reason, during the lockdown period medical doctors recommended the consumption of foods typical of the MD because they could play a therapeutic role in conditions related to COVID-19 infection [36]. As regard the changes in the diet during the pandemic period, although there was a daily increase in the frequencies of butter, cream and margarine use, mainly caused by the preparation of homemade products [9], consumption of sweet/carbonated beverages, and an increase in the servings of sweets consumed weekly, the significant increase in the consumption of healthy food such as fruits and vegetables, legumes and nuts, the consumption of wine, seasoned dishes with sofrito and the reduction in red meat consumption determined the increase in adherence to the MD. Important result emerging from our study, contrary to the findings shown by Caso D. [37], these eating habits are not only maintained, they also improve after the gradual return to normal life. Our sample study shows a positive trend towards a plant-based dietary pattern that has been shown to be a powerful tool to decrease the risk of severe Covid-19 and mortality [38]. After the lockdown, there appears to be an increase in the importance attributed to health, weight control, and weight itself as motivations for food choices, as indicated by recent findings by Snuggs & McGregor [39]. In this regard, our results show a reduction of red meat consumption, and this is in line with the results of Gerini F et al., Grant et al., [11, 40]. Habit adopted by more and more people because of the increased concern of human health, but especially environmental health [41]. During the pandemic period, although one of the mainstays of the Mediterranean model - conviviality - was missing, traditional plates, recipes and authentic flavours were rediscovered. At the same time it was found that people spent more time preparing meals, which probably helped people relieve stress [42] and distract themselves from the pandemic, in fact culinary activities were defined as an “escape” during COVID-19 by Güler, O., & Haseki, M. İ. [43], especially for people who were suddenly faced with a closure that almost no one was accustomed to. Consistent with meta-analysis of Bizzozero-Peroni, Bruno et al. [44] the results of our study show a positive association between adherence to the MD and PA. MD was positively correlated with PA levels in pre-pandemic (p < 0.001, r = 0.176) during the pandemic (p < 0.001, r = 0.182) post-pandemic (p < 0.001, r = 0.154).

Thus, although a better quality of dietary habits has emerged, there is probably an excess of calories due to high consumption of high energy density food [45] and lower energy expenditure due to the increased time spent in sedentary mode and the drastic reduction of PA, which have caused an inevitable increase in BMI in the population examined.

The results of this study showed a significant increase in BMI from a normal weight condition in the pre-pandemic period to an overweight condition in the post-pandemic period. This result is in agreement with previous research that analysed differences in BMI before and during the pandemic [24, 46] which found a significant increase in BMI that could be attributed to an increase in caloric intake and an overall reduction in PA levels due to the isolation of COVID-19. Brancaccio et al, [16] reported that although there was an increase in BMI of 0.2 Kg, participants maintained a normal weight status. In our study, no differences were found in BMI between genders, according to results of Almousa and Alagal [26]. However, this outcome is contrary to that of Brancaccio et al., [16] who found a significant difference in BMI between male and female in both pre-pandemic and post-pandemic periods.

## Strengths and limitations

This study has several notable strengths. One of the primary is its large sample size, which included 2773 adults from various regions of Italy, providing a comprehensive and representative view of the Italian population’s lifestyle changes. Another significant strength is the longitudinal approach adopted by the researchers, which allowed for the examination of three distinct periods: pre-pandemic, during the pandemic, and post-pandemic. This approach effectively captures both the short-term and long-term impacts of the pandemic on physical activity and dietary habits. Additionally, the use of validated instruments such as IPAQ-SF and MEDAS ensured that the data collected were reliable and valid. The diverse recruitment methods, including social media and word-of-mouth, also contributed to the robustness of the sample by reaching a wide audience.

However, the study also has limitations. The reliance on self-reported data for physical activity, dietary habits, and anthropometric measures could introduce self-report bias, as participants might overestimate or underestimate their behaviors and characteristics. Furthermore, the cross-sectional design of the survey administered in a single post-pandemic period limits the ability to make causal inferences about the observed changes over time. The study population was predominantly female, which might affect the generalizability of the findings to the entire Italian population. Lastly, the study did not account for all potential confounding factors such as psychological stress and economic status, which could influence changes in lifestyle behaviors during the pandemic.

## Conclusions

This study provides information on the PA, sedentary lifestyle, eating behaviour and adherence to the Mediterranean dietary pattern of the Italian population before, during and after the pandemic period. The lockdown invariably changed people’s lifestyles, but in different ways for PA and diet. During the pandemic there was a negative effect for PA that decreased while the time spent sitting increased. This seems to be a temporary effect as, after the end of the phase of mandatory restrictions, it returned to the original level. In terms of diet, there were positive effects: a rediscovery of traditional dishes, increase in consumption of seasonal products, greater preference for “zero-km-products” and more time spent preparing lunch and dinner. Moreover, during the pandemic period there was an increased consumption of healthy, mainly plant-based foods (fruits, vegetables, legumes, nuts) which has contributed to a “moderate” adherence to the MD. The lockdown period thereby improved the quality of the Italian population’s eating habits, so much so that adherence to the MD continues to increase even after the end of the pandemic, but it certainly also affects the quantity of food consumed, as there is an increase in BMI. Further investigations would be useful to know the current adherence to the MD and participation in PA as useful strategies to reduce the body weight gained during the mandatory lockdown period.

## Data Availability

Data have been organized in an Excel file, which is kept by the investigators and it will become available upon request.

## References

[1] World Health Organization. Non-pharmaceutical public health measures for mitigating the risk and impact of epidemic and pandemic influenza [Internet]. Geneva: World Health Organization; 2019. 85 p. Disponibile su: https://iris.who.int/handle/10665/329438

[2] S. Flaxman, S. Mishra, A. Gandy, H.J.T. Unwin, T.A. Mellan, H. Coupland et al. Estimating the effects of non-pharmaceutical interventions on COVID-19 in Europe. Nature **584**(agosto 7820), 257–261 (2020). 10.1038/s41586-020-2405-7 32512579 10.1038/s41586-020-2405-7

[3] D. Kaur, P. Rasane, J. Singh, S. Kaur, V. Kumar, D.K. Mahato et al. Nutritional Interventions for Elderly and Considerations for the Development of Geriatric Foods. Curr. Aging Sci. **12**(settembre 1), 15–27 (2019). 10.1007/s10668-020-00884-x 31109282 10.2174/1874609812666190521110548 PMC6971894

[4] A. Catucci, U. Scognamiglio, L. Rossi, Lifestyle Changes Related to Eating Habits, Physical Activity, and Weight Status During COVID-19 Quarantine in Italy and Some European Countries. Front Nutr. **8**(agosto), 718877 (2021). 10.3389/fnut.2021.718877 34490330 10.3389/fnut.2021.718877 PMC8417694

[5] L. Timelli, E. Girardi, Effect of timing of implementation of containment measures on Covid-19 epidemic. case first wave Italy Lolli S, Curator. PLOS One. **16**(gennaio 1), e0245656 (2021). 10.1371/journal.pone.0245656 10.1371/journal.pone.0245656 PMC7846280 33513157

[6] M. Lombardo, E. Guseva, M.A. Perrone, A. Müller, G. Rizzo, M.A. Storz, Changes in Eating Habits and Physical Activity after COVID-19 Pandemic Lockdowns in Italy. Nutrients **13**(dicembre 12), 4522 (2021). 10.3390/nu13124522 34960077 10.3390/nu13124522 PMC8708956

[7] T. Galanti, G. Guidetti, E. Mazzei, S. Zappalà, F. Toscano, Work From Home During the COVID-19 Outbreak: The Impact on Employees’ Remote Work Productivity, Engagement, and Stress. J. Occup. Environ. Med **63**(7), e426–e432 (2021). 10.1097/JOM.0000000000002236. 33883531 10.1097/JOM.0000000000002236 PMC8247534

[8] L. Cardinali, D. Curzi, E. Maccarani, L. Falcioni, M. Campanella, D. Ferrari et al. Live Streaming vs. Pre-Recorded Training during the COVID-19 Pandemic in Italian Rhythmic Gymnastics. Int J. Environ. Res Public Health **19**(dicembre 24), 16441 (2022). 10.3390/ijerph192416441 36554324 10.3390/ijerph192416441 PMC9778436

[9] L. Di Renzo, P. Gualtieri, F. Pivari, L. Soldati, A. Attinà, G. Cinelli et al. Eating habits and lifestyle changes during COVID-19 lockdown: an Italian survey. J. Transl. Med **18**(dicembre 1), 229 (2020). 10.1186/s12967-020-02399-5 32513197 10.1186/s12967-020-02399-5 PMC7278251

[10] F. Farrugia, D. Refalo, D. Bonello, S. Cuschieri, The impact of the COVID-19 pandemic on Mediterranean diet adherence: A narrative systematic review. Nutr Health. luglio;02601060231187511. 10.1177/02601060231187511 (2023) 10.1177/02601060231187511 PMC10345400 37439029

[11] F. Grant, M.L. Scalvedi, U. Scognamiglio, A. Turrini, L. Rossi, Eating Habits during the COVID-19 Lockdown in Italy: The Nutritional and Lifestyle Side Effects of the Pandemic. Nutrients **13**(7), 2279 (2021). 10.3390/nu13072279. 34209271 10.3390/nu13072279 PMC8308479

[12] K. Orr, Z. Ta, K. Shoaf, T.M. Halliday, S. Tobin, K.G. Baron, Sleep, Diet, Physical Activity, and Stress during the COVID-19 Pandemic: A Qualitative Analysis. Behav. Sci. **12**(3), 66 (2022). 10.3390/bs12030066. 35323385 10.3390/bs12030066 PMC8945701

[13] L.M. Donini, L. Serra-Majem, M. Bulló, Á. Gil, J. Salas-Salvadó, The Mediterranean diet: culture, health and science. Br. J. Nutr. **113**(aprile S2), S1–S3 (2015). 10.1017/S0007114515001087 26148911 10.1017/S0007114515001087

[14] E. Franco, J. Urosa, R. Barakat, I. Refoyo, Physical Activity and Adherence to the Mediterranean Diet among Spanish Employees in a Health-Promotion Program before and during the COVID-19 Pandemic: The Sanitas-Healthy Cities Challenge. Int J. Environ. Res Public Health **18**(5), 2735 (2021). 10.3390/ijerph18052735 33800372 10.3390/ijerph18052735 PMC7967464

[15] A. Ammar, M. Brach, K. Trabelsi, H. Chtourou, O. Boukhris, L. Masmoudi et al. Effects of COVID-19 Home Confinement on Eating Behaviour and Physical Activity: Results of the ECLB-COVID19 International Online Survey. Nutrients **12**(6), 1583 (2020). 10.3390/nu12061583. 32481594 10.3390/nu12061583 PMC7352706

[16] M. Brancaccio, C. Mennitti, A. Gentile, L. Correale, C.F. Buzzachera, C. Ferraris, et al. Effects of the COVID-19 Pandemic on Job Activity, Dietary Behaviours and Physical Activity Habits of University Population of Naples, Federico II-Italy. Int J. Environ. Res Public Health **18**(4), 1502 (2021). 10.3390/ijerph18041502 33562476 10.3390/ijerph18041502 PMC7915794

[17] S. De Nucci, R. Zupo, F. Castellana, A. Sila, V. Triggiani, G. Lisco, G. De Pergola, R. Sardone, Public Health Response to the SARS-CoV-2 Pandemic: Concern about Ultra-Processed Food Consumption. Foods **11**(Mar 7), 950 (2022). 10.3390/foods11070950 35407037 10.3390/foods11070950 PMC8997472

[18] R. Zupo, F. Castellana, R. Sardone, A. Sila, V.A. Giagulli, V. Triggiani, R.I. Cincione, G. Giannelli, G. De Pergola, Preliminary Trajectories in Dietary Behaviors during the COVID-19 Pandemic: A Public Health Call to Action to Face Obesity. Int J. Environ. Res Public Health **17**(Sep 19), 7073 (2020). 10.3390/ijerph17197073 32992623 10.3390/ijerph17197073 PMC7579065

[19] L. Bertocchi, R. Vecchio, S. Sorbello, L. Gentile, M. Gaeta, A. Odone, Impact of the COVID-19 pandemic on physical activity among university students in Pavia, Northern Italy. Acta Biomed. Atenei Parm. **92**(dicembre S6), e2021443 (2021). 10.23750/abm.v92iS6.12232 10.23750/abm.v92iS6.12232 PMC8851014 34889314

[20] M. Guicciardi, R. Pazzona, The Rebooting in Sports and Physical Activities After COVID-19 Italian Lockdown: An Exploratory Study. Front Psychol. **11**(novembre), 607233 (2020). 10.3389/fpsyg.2020.607233 33324304 10.3389/fpsyg.2020.607233 PMC7723834

[21] S.E. Schöttl, M. Schnitzer, L. Savoia, M. Kopp, Physical Activity Behavior During and After COVID-19 Stay-at-Home Orders—A Longitudinal Study in the Austrian, German, and Italian Alps. Front Public Health **10**, 901763 (2022). 10.3389/fpubh.2022.901763. 35712287 10.3389/fpubh.2022.901763 PMC9194442

[22] C.L. Craig, A.L. Marshall, M. Sjöström, A.E. Bauman, M.L. Booth, B.E. Ainsworth et al. International Physical Activity Questionnaire: 12-Country Reliability and Validity: Med Sci Sports Exerc. Med Sci. Sports Exerc **35**(8), 1381–1395 (2003). 10.1249/01.MSS.0000078924.61453.FB. 12900694 10.1249/01.MSS.0000078924.61453.FB

[23] I. Zazpe, A. Sanchez-Tainta, R. Estruch, R.M. Lamuela-Raventos, H. Schröder, J. Salas-Salvado et al. A Large Randomized Individual and Group Intervention Conducted by Registered Dietitians Increased Adherence to Mediterranean-Type Diets: The PREDIMED Study. J. Am. Diet. Assoc. **108**(7), 1134–1144 (2008). 10.1016/j.jada.2008.04.011. 18589019 10.1016/j.jada.2008.04.011

[24] F. Branca, H. Nikogosian, T. Lobstein, World Health Organization, curatori. The challenge of obesity in the WHO European Region and the strategies for response (WHO Regional Office for Europe, Copenhagen, Denmark, 2007), p. 323. https://iris.who.int/bitstream/handle/10665/326533/9789289014083-eng.pdf

[25] J.W. Ripley-Gonzalez, N. Zhou, T. Zeng, B. You, W. Zhang, J. Liu et al. The long-term impact of the COVID-19 pandemic on physical fitness in young adults: a historical control study. Sci. Rep. **13**(settembre 1), 15430 (2023). 10.1038/s41598-023-42710-0 37723197 10.1038/s41598-023-42710-0 PMC10507106

[26] L.A. Almousa, R.I. Alagal, Effects of the COVID-19 pandemic on diet and physical activity and the possible influence factors among Saudi in Riyadh. Front Nutr. **9**, 1029744 (2022). 10.3389/fnut.2022.1029744. 36337667 10.3389/fnut.2022.1029744 PMC9630832

[27] W.R. Thompson, Worldwide Survey of Fitness Trends for 2021. ACSMS Health Fit. J. gennaio **25**(1), 10–19 (2021). 10.3389/fnut.2022.1029744. DOI: 10.1249/FIT.0000000000000631

[28] A.J. Holmes, T.D. Quinn, M.B. Conroy, J.L. Paley, K.A. Huber, B. Barone Gibbs, Associations of Physical and Social Workplace Characteristics with Movement Behaviors at Work. Transl. J. Am. Coll. Sports Med [Internet] **8**(2), e000225 (2023). 10.1249/TJX.0000000000000225. 36819009 10.1249/tjx.0000000000000225 PMC9937511

[29] P. De Oliveira Da Silva Scaranni, R.H. Griep, F.J.G. Pitanga, S.M. Barreto, S.M.A. Matos, M. De Jesus Mendes Da Fonseca, Work from home and the association with sedentary behaviors, leisure-time and domestic physical activity in the ELSA-Brasil study. BMC Public Health **23**(1), 305 (2023). 10.1186/s12889-023-15167-z. febbraio 36765304 10.1186/s12889-023-15167-z PMC9912204

[30] F. Qin, Y. Song, G.P. Nassis, L. Zhao, Y. Dong, C. Zhao et al. Physical Activity, Screen Time, and Emotional Well-Being during the 2019 Novel Coronavirus Outbreak in China. Int J. Environ. Res Public Health **17**(14), 5170 (2020). 10.3390/ijerph17145170. 32709003 10.3390/ijerph17145170 PMC7399902

[31] F. Bu, J.K. Bone, J.J. Mitchell, A. Steptoe, D. Fancourt, Longitudinal changes in physical activity during and after the first national lockdown due to the COVID-19 pandemic in England. Sci. Rep. **11**(settembre 1), 17723 (2021). 10.1038/s41598-021-97065-1 34475465 10.1038/s41598-021-97065-1 PMC8413348

[32] L. Izzo, A. Santonastaso, G. Cotticelli, A. Federico, S. Pacifico, L. Castaldo et al. An Italian Survey on Dietary Habits and Changes during the COVID-19 Lockdown. Nutrients **13**(aprile 4), 1197 (2021). 10.3390/nu13041197 33916384 10.3390/nu13041197 PMC8065756

[33] J.L. Grantham, C.L. Verishagen, S.J. Whiting, C.J. Henry, J.R.L. Lieffers, Evaluation of a Social Media Campaign in Saskatchewan to Promote Healthy Eating During the COVID-19 Pandemic: Social Media Analysis and Qualitative Interview Study. J. Med Internet Res **23**(7), e27448 (2021). 10.2196/27448. 34133314 10.2196/27448 PMC8297600

[34] Dernini S., Berry E.M. Historical and Behavioral Perspectives of the Mediterranean Diet. In: Romagnolo D.F., Selmin O.I., curatori. Mediterranean Diet [Internet]. Cham: Springer International Publishing; 2016. p. 29–41. Disponibile su: http://link.springer.com/10.1007/978-3-319-27969-5_3

[35] L. Barrea, G. Muscogiuri, E. Frias-Toral, D. Laudisio, G. Pugliese, B. Castellucci et al. Nutrition and immune system: from the Mediterranean diet to dietary supplementary through the microbiota. Crit. Rev. Food Sci. Nutr. **61**(18), 3066–3090 (2021). 10.1080/10408398.2020.1792826. 32691606 10.1080/10408398.2020.1792826

[36] A.M. Angelidi, A. Kokkinos, E. Katechaki, E. Ros, C.S. Mantzoros, Mediterranean diet as a nutritional approach for COVID-19. Metabolism **114**, 154407 (2021). 10.1016/j.metabol.2020.154407. 33080270 10.1016/j.metabol.2020.154407 PMC7833284

[37] D. Caso, M. Guidetti, M. Capasso, N. Cavazza, Finally, the chance to eat healthily: Longitudinal study about food consumption during and after the first COVID-19 lockdown in Italy. Food Qual. Prefer **95**, 104275 (2022). 10.1016/j.foodqual.2021.104275. 34539093 10.1016/j.foodqual.2021.104275 PMC8443069

[38] H. Kahleova, N.D. Barnard, Can a plant-based diet help mitigate Covid-19? Eur. J. Clin. Nutr. **76**(7), 911–912 (2022). 10.1038/s41430-022-01082-w. 35064220 10.1038/s41430-022-01082-w PMC8777176

[39] S. Snuggs, S. McGregor, Food & meal decision making in lockdown: How and who has Covid-19 affected? Food Qual. Prefer. **89**(aprile), 104145 (2021). 10.1016/j.foodqual.2020.104145 33250586 10.1016/j.foodqual.2020.104145 PMC7685931

[40] F. Gerini, T. Fantechi, C. Contini, L. Casini, G. Scozzafava, Adherence to the Mediterranean Diet and COVID-19: A Segmentation Analysis of Italian and US Consumers. Sustainability **14**(7), 3823 (2022). 10.3390/su14073823.

[41] F. Bert, M. Gea, C. Previti, G. Massocco, G. Lo Moro, G. Scaioli et al. The Environmental Health Literacy of Italian General Population: The SPeRA Cross-Sectional Study. Int J. Environ. Res Public Health **20**(5), 4486 (2023). 10.3390/ijerph20054486. 36901494 10.3390/ijerph20054486 PMC10002404

[42] W. Shen, L.M. Long, C.H. Shih, M.J. Ludy, A Humanities-Based Explanation for the Effects of Emotional Eating and Perceived Stress on Food Choice Motives during the COVID-19 Pandemic. Nutrients **12**(settembre 9), 2712 (2020). 10.3390/nu12092712 32899861 10.3390/nu12092712 PMC7551550

[43] O. Güler, M.İ. Haseki, Positive Psychological Impacts of Cooking During the COVID-19 Lockdown Period: A Qualitative Study. Front Psychol. **12**, 635957 (2021). 10.3389/fpsyg.2021.635957 33815223 10.3389/fpsyg.2021.635957 PMC8012501

[44] B. Bizzozero-Peroni, J. Brazo-Sayavera, V. Martínez-Vizcaíno, R. Fernández-Rodríguez, J.F. López-Gil, V. Díaz-Goñi et al. High Adherence to the Mediterranean Diet is Associated with Higher Physical Fitness in Adults: a Systematic Review and Meta-Analysis. Adv. Nutr. **13**(6), 2195–2206 (2022). 10.1093/advances/nmac104 36166848 10.1093/advances/nmac104 PMC9776663

[45] N.J. Buckland, E. Kemps, Low craving control predicts increased high energy density food intake during the COVID-19 lockdown: Result replicated in an Australian sample. Appetite **166**(novembre), 105317 (2021). 10.1016/j.appet.2021.105317 34048847 10.1016/j.appet.2021.105317 PMC9756090

[46] S. Maffoni, S. Brazzo, R. De Giuseppe, G. Biino, I. Vietti, C. Pallavicini et al. Lifestyle Changes and Body Mass Index during COVID-19 Pandemic Lockdown: An Italian Online-Survey. Nutrients **13**(4), 1117 (2021). 10.3390/nu13041117. 33805291 10.3390/nu13041117 PMC8066204

